# Degradation intermediates of polyhydroxy butyrate inhibits phenotypic expression of virulence factors and biofilm formation in luminescent *Vibrio* sp. PUGSK8

**DOI:** 10.1038/npjbiofilms.2016.2

**Published:** 2016-06-15

**Authors:** George Seghal Kiran, Sethu Priyadharshini, Alan D W Dobson, Elumalai Gnanamani, Joseph Selvin

**Affiliations:** 1 Department of Food Science and Technology, Pondicherry University, Puducherry, India; 2 School of Microbiology, University College Cork, Cork, Ireland; 3 Environmental Research Institute, University College Cork, Cork, Ireland; 4 Department of Chemistry, Stanford University, Stanford, CA, USA; 5 Department of Microbiology, School of Life Sciences, Pondicherry University, Puducherry, India

## Abstract

Luminescent vibrios are ubiquitous in the marine environment and are the causative agents of vibriosis and mass mortality in many aquatic animals. In aquatic environments, treatments cannot be limited to the diseased population alone, therefore treatment of the entire aquatic system is the only possible approach. Thus, the use of antibiotics to treat part of the infected animals requires a dose based on the entire biomass, which results in the treatment of uninfected animals as well as non-target normal microbial flora. A treatment method based on anti-virulence or quorum quenching has recently been proposed as an effective treatment strategy for aquatic animals. Polyhydroxy butyrates (PHB) are bacterial storage molecules, which accumulate in cells under nutritional stress. The degradation of PHB releases short-chain β-hydroxy butyric acid, which may act as anti-infective molecule. To date, there is very limited information on the potential anti-infective and anti-virulence mechanisms involving PHB. In this study, we aim to examine the effect of PHB on inhibition of the virulence cascade of *Vibrio* such as biofilm formation, luminescence, motility behaviour, haemolysin and quorum sensing. A luminescent *Vibrio* PUGSK8, tentatively identified as *Vibrio campbellii* PUGSK8 was tested *in vitro* for production of extracellular virulence factors and then established as a potential shrimp pathogen based on *in vivo* challenge experiments. The ability of *Vibrio* PUGSK8 to form biofilms and the effect of PHB on biofilm formation was tested in a 96-well microtitre-plate assay system. The motility behaviour of *Vibrio* PUGSK8 was evaluated using twitching, swimming and swarming plate assays. Reporter strains such as *Chromobacterium violaceum* CV026 and *Agrobacterium tumefaciens* were used to detect quorum-sensing molecules. Gas chromatography–mass spectrometry spectral analysis was performed to elucidate the fragmentation pattern and structure of *N*-hexanoyl homoserine lactone. PHB depolymerase activity in *Vibrio* PUGSK8 was quantified as the amount of the enzyme solution to hydrolyse 1 μg of PHB per min. An *in vivo* challenge experiment was performed using a gnotobiotic Artemia assay. Of the 27 isolates tested, the *Vibrio* PUGSK8 strain was selected for target-specific assays based on the high intensity of luminescence and production of virulence factors. The virulence cascade detected in *Vibrio* PUGSK8 include luminescence, motility behaviour, biofilm formation, quorum sensing and haemolysin production. Thus inhibition/degradation of the virulence cascade would be an effective approach to contain *Vibrio* infections in aquatic animals. In this report, we demonstrate that the degradation intermediate of PHB effectively inhibits biofilm formation, luminescence, motility behaviour, haemolysin production and the *N*-acyl-homoserine lactone (AHL)-mediated quorum-sensing pathway in PUGSK8. Interestingly, the growth of *Vibrio* PUGSK8 remains unaffected in the presence of PHB, with PHB degradation being detected in the media. PHB depolymerase activity in *Vibrio* PUGSK8 results in the release of degradation intermediates include a short-chain β-hydroxy butyric acid, which inhibits the virulence cascade in *Vibrio* PUGSK8. Thus, a molecule that targets quorum sensing and the virulence cascade and which is species/strain-specific could prove to be an effective alternative to antimicrobial agents to control the pathogenesis of *Vibrio*, and thereby help to contain *Vibrio* outbreaks in aquatic systems.

## Introduction

Member of the *Vibrio* genus are common inhabitants of various aquatic environments. They typically exist either as free-living organisms or associated with hosts such as zooplankton, which are known to protect the *Vibrio* from a variety of different environmental stresses. *Vibrio* have also been reported to be associated with many higher organisms in marine environments including corals, crabs, molluscs and fish among others. Although the majority of these associations are not harmful to the host, there are examples where *Vibrio* spp. are pathogenic with *Vibrio coralliilyticus* and *Vibrio tubiashii* being the causative agents of disease in commercially important organisms such as oysters and corals, respectively. Others include the luminescent strain *Vibrio harveyi*, which is the potential causative agent of mass mortality in shrimp aquaculture worldwide,^1^ together with *Vibrio harveyi* and *Vibrio alginolyticus* which are the most common pathogens of giant black tiger shrimp *Penaeus monodon* in Asia and pose the principal threat faced by shrimp hatcheries all over the world. *Vibrio parahaemolyticus* is believed to be the primary causative agent of the recent mass mortality in shrimp due to early-mortality syndrome.^2^ The biofilm-forming capacity of *V. cholerae* is well documented, both in natural habitats and under laboratory conditions.^3–5^ Among the shrimp *Vibrio* pathogens, the biofilm-forming capacity of *V. harveyi* has been established on cement slab, plastic and steel coupon surfaces.^6^ Adhesion and proliferation within the biofilm is an established mechanism of pathogenesis and infection of *V. harveyi* in *P. monodon.*^7^ In addition several studies have suggested that biofilms are important for survival, virulence and stress resistance in *Vibrio* spp.,^4,8–12^ with biofilm formation being commonly associated with colonisation and subsequent pathogenesis in hosts by vibrios in marine environments.^13^ To date, only a few studies have been carried out on biofilm inhibition in *Vibrio* spp.^14–16^ Bacteria in biofilms are surrounded by an extracellular matrix that can restrict diffusion of antimicrobial agents.^17^ In addition changes in the membrane sterol composition in bacteria during biofilm development can also increase the microbial cell’s resistant to antibiotics.^18,19^

Quorum sensing is a cell-to-cell communication process in bacteria that involves the production, release, detection and collective response to extracellular signal molecules called autoinducers, which control the phenotypic expression of bioluminescence, biomass development, ecological succession, competence, biofilm formation, motility and the production of virulence factors.^20,21^ Gram-negative quorum-sensing circuits rely on *LuxI* dependent acyl homoserine lactones (AHL) and a LuxR-type autoinducer binding transcriptional regulator protein. The quorum-sensing circuit of *V. harveyi* is known to consist of a three-channel model signal transduction pathway. The first channel is mediated by acylated homoserine lactone-autoinducer1 (HAI-1), the second channel by a furanosyl borate diester—autoinducer-2 and the third by a cholera autoinducer-1 (CAI-1).^22–24^ Updates on the taxonomic revisions of *Vibrio* clades are still inconclusive, and require comprehensive revisions particularly with respect to luminescent *Vibrio* clades. For instance luminescent *V. campbellii* belonging to the *V. harveyi* clade are known to use the quorum-sensing molecule *N*-hexanoyl homoserine lactone.^25,26^ The quorum-sensing molecules identified in luminescent vibrios include C6 homoserine lactone in *V. fischeri* and C4 homoserine lactone in *V. harveyi*. But there is little conclusive information on quorum-sensing molecules produced in various other species in the *V. harveyi* clade. Given that quorum sensing is a molecular mechanism involved in the expression of virulence factors in many pathogenic bacteria, then interference with quorum sensing and alteration of quorum sensing circuits can effectively regulate virulence expression and pathogenicity.

Polyhydroxy butyrate (PHB) is a common bacterial intracellular biopolymer that appear as granules and are produced in bacterial cells under nutritional limitation or when they are in environments, which are unsuitable for cell growth. The PHB polymer can be hydrolysed to short-chain β-hydroxy butyric acid, which has been shown to be an effective anti-infective molecule in the gastrointestinal tract of the shrimp providing 73% protection to treated animal.^27,28^ PHB can also act as an inducer of HSP70, which provide protection against *V. campbelli* infection in Artemia.^29^ However, the mechanism of action of PHB and any potential effect that it may exert on quorum sensing/quenching has to date not been fully established. On the basis of current reports in the literature, potential mechanisms for the anti-infective effect of PHB may occur as a result of either (i) PHB being hydrolysed to 3-hydroxy butyric acid by the digestive enzymes present in the gastrointestinal tract of the treated animal or (ii) owing to the PHB depolymerase activity of resident bacteria in the gut. In this paper, we report that the degradation intermediates of PHB include β-hydroxy butyric acid which exhibits antibacterial activity against *Vibrio* PUGSK8 in both *in vitro* plate assay and *in vivo* challenge experiments in a gnotobiotic Artemia model. The shrimp pathogen *Vibrio* PUGSK8 showed PHB depolymerase activity, resulting in the production of a PHB degradation intermediate, which effectively shuts down the phenotypic expression of virulence factors and biofilm formation. Interestingly, the PHB degradation intermediate does not appear to have a detrimental effect on the growth of *Vibrio* PUGSK8 resulting in the microbial biomass remaining unaffected. In this study, we report on the anti-infective effect of PHB, which inhibits the virulence cascade in *Vibrio* PUGSK8. The degradation intermediate of PHB reduces motility mediated by flagellar and pili adhesion factors resulting in the disruption of biofilm formation, and inhibited phenotypic expression of bioluminescence, haemolysin and quorum-sensing mediated through AHL.

## Results

### Identification and characterisation of pathogenic *Vibrio* PUGSK8

**Figure 1:**
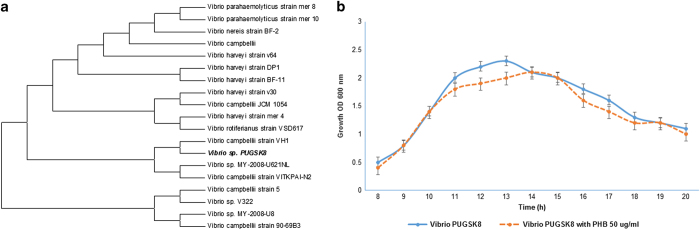
Phylogenetic and growth characteristics of *Vibrio* PUGSK8. (**a**) Phylogenetic tree of *Vibrio* sp. PUGSK8. Maximum parsimony consensus phylogenetic tree constructed using MEGA 6.0 based on 16S rRNA gene sequence of *Vibrio* sp. PUGSK8 showing representatives of other related taxa. The phylogenetic analysis showed 99% to *Vibrio harveyi* and *Vibrio campbellii*. On the basis of the AHL molecule produced by the strain PUSK8, it was tentatively identified as *Vibrio campbellii* strain PUGSK8. (**b**) The effect of PHB on growth of *Vibrio* PUGSK8. The growth was recorded at OD_600 nm_. The isolate (control) entered logarithmic phase after 9 h of growth and started to decline after 17 h. The trend of growth pattern was not affected in the media supplemented with PHB (50 μg/ml).

Bacterial isolates were obtained from diseased shrimp samples collected from a shrimp hatchery located on the southeast coast of India. Among the 68 colonies, the isolates were grouped into luminescent and non-luminescent bacteria. All 27 luminescent colonies showed *in vitro* expression of virulence factors such as phospholipase and haemolysin (Supplementary Table S1). Among these, the strain PUGSK8 was chosen as it displayed very high levels of luminescence an indirect factor, which is produced as part of the virulence cascade. Strain PUGSK8 was tentatively identified as *Vibrio campbelli* based on biochemical and phylogenetic analysis. The strain PUGSK8 was sensitive to O/129 and tested positive for a number of extracellular virulence factors including phospholipase, haemolysin, elastase, chitinase and cell surface hydrophobicity. Phylogenetic analysis of the PUGSK8 16S ribosomal RNA (rRNA) sequence showed closet matches of 99% with *Vibrio campbelli* belonging to the *Vibrio harveyi* clade (Figure 1a). The sequence data were submitted to Genbank with the accession number KR024645. *V. campbellii* PUGSK8 formed biofilm growth on various surfaces such as glass, polystyrene and plastic (Supplementary Figure S1). The strain was further tested *in vivo* to establish its pathogenicity to cause shell disease in healthy challenged shrimps. Strain PUGSK8 was established as a potential shrimp pathogen based on challenge experiments (Supplementary Figure S2).

### Bacterial growth and luminescence

**Figure 2:**
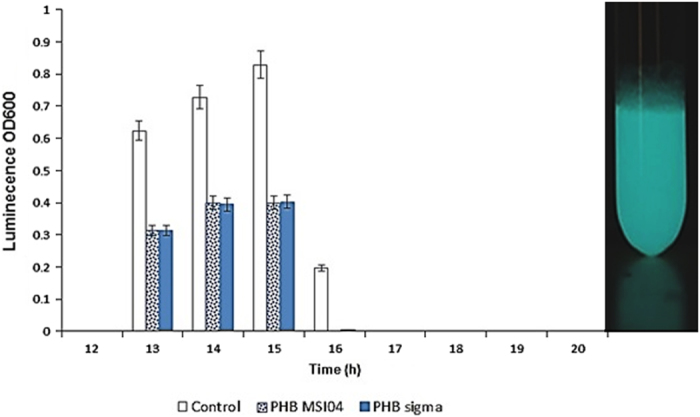
The intensity of luminescence during the growth cycle of *Vibrio* PUGSK8. (**a**) The intensity of luminescence decreased in the media supplemented with PHB (MSI04) and PHB (sigma). The luminescence reached detectable levels at the 13^th^ h of incubation and production became undetectable after 16th of incubation. (**b**) Luminescence in *Vibrio* PUGSK8 following 13h incubation.

Given that bioluminescence in *Vibrio* species is one of the phenotypes which is controlled by quorum sensing, we examined the possibility that PHB may affect bioluminescence in *Vibrio* PUGSK8. Initial experiments were conducted to determine that the addition of PHB (50 μg/ml) to *Vibrio* PUGSK8 cultures did not have a detrimental effect on cell growth (Figure 1b). The growth profiles for PUGSK8 grown in the presence and absence of PHB were similar, with cultures entering logarithmic phase after 9 h, with decreased growth being observed after 17 h. Thus, no detrimental effect on the growth of *Vibrio* PUGSK8 was apparent. However, when bioluminescence was measured between 13 and 15 h following incubation high levels of bioluminescence were observed in the control, whereas in the PHB-treated (50 μg/ml) flasks reduced levels of bioluminescence were observed, in both PHB-treated cultures such as PHB MSI04 and PHB standard (Sigma-Aldrich Corporation, Bangalore, India) (Figure 2). At 16 h, bioluminescence in the control *Vibrio* PUGSK8 cultures had decreased markedly, while no bioluminescence was observed in the PHB cultures. Thus, given that PHB appears to inhibit bioluminescence in *Vibrio* PUGSK8 cultures, we then examined the possibility that this inhibition may affect the production of various virulence factors, which like bioluminescence are known to be regulated by quorum sensing in *Vibrio*.

### Inhibition of motility and biofilm formation

**Figure 3:**
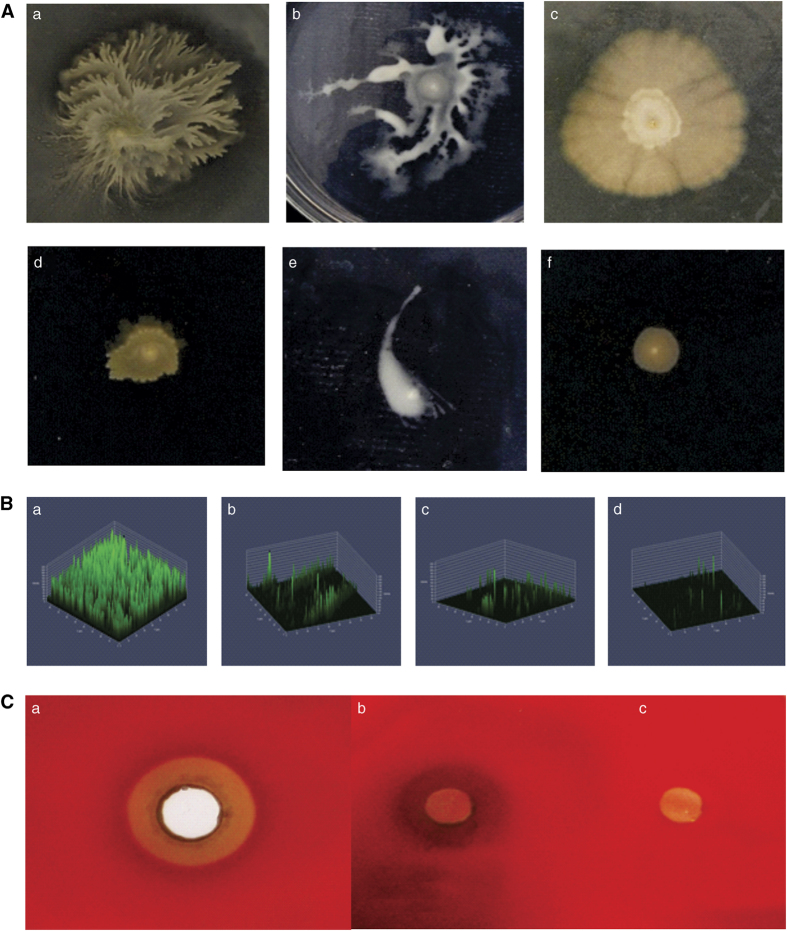
The effect of degradation intermediates of PHB on the phenotypic expression of virulence factors. (**A**) The effect of PHB on the motility behaviour of *Vibrio* PUGSK8. The PHB treatment effected the loss of twitching, swarming and swimming behaviours. The effect of PHB on twitching (a–control, d—treated), swarming (b—control, e—treated) and swimming (c—control, f—treated). (**B**) CLSM images shows the effect PHB on biofilm formation by *Vibrio* PUGSK8. The *a* is control and *b, c* and *d* is showing the effect of PHB on biofilm formation at concentrations of 50, 100 and 150 μg/ml, respectively. (**C**) Haemolytic activity of *Vibrio* PUGSK8 on blood agar plate. (*a*) The cell-free supernatant (CFS) of untreated *Vibrio* PUGSK8 showing haemolytic activity (*b*) Haemolytic activity of CFS collected from PHB-treated *Vibrio* (24 h) (*c*) Complete inactivation of haemolytic activity of CFS collected from PHB-treated *Vibrio* (48 h).

Motility is an important virulence factor in the virulence cascade of *V. campbelli*, as it contributes to biofilm formation. *Vibrio* PUGSK8 displayed pronounced flagellar and pili mediated motility as evident by the twitching, swimming and swarming assays (Figure 3A). The addition of PHB (50 μg/ml), however, inhibited the motility of PUGSK8 which in turn reduced the colonisation capacity of the strain on various tested surfaces. PHB completely inhibited the swimming, swarming and twitching motility of *Vibrio* PUGSK8, whereas a clear effect on biofilm formation was also evident from the microtitre-plate assay and confocal laser scanning microscopy (CLSM) image analysis (Figure 3B), with a concentration of 150 μg PHB being the most effective in reducing the biofilm formation. Given that PUGSK8 can effectively colonise various surfaces such as glass, polystyrene and plastic (Figure 1c), it appears likely that the observed inhibition of motility may result in reducing the adhesion, and colonisation capacity of *Vibrio* PUGSK08.

### Haemolytic and PHB depolymerase activity of *Vibrio* PUGSK8

**Figure 4:**
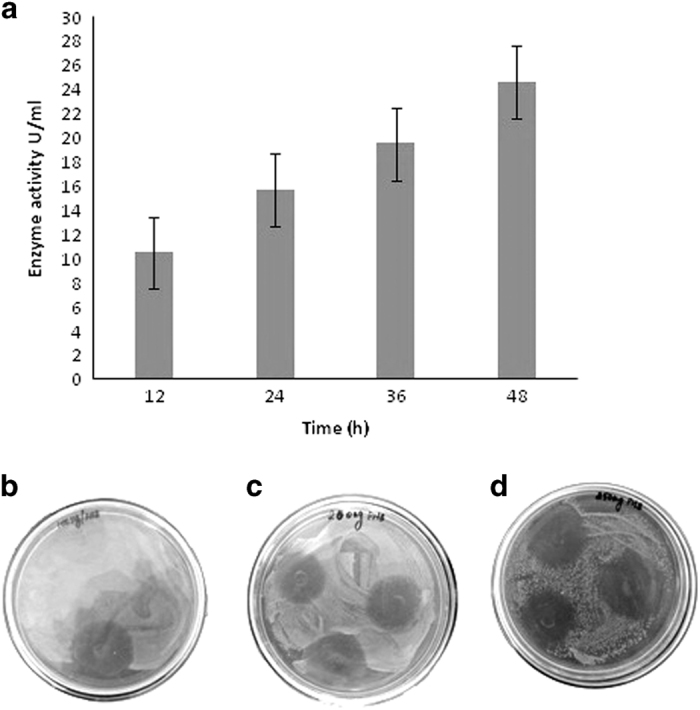
PHB depolymerase produed by *Vibrio* PUGSK8 at various time intervals. (**a**) is showing PHB depolymerase activity in U/ml. The PHB depolymerase activity on minimal media supplemented with 100 mg/l PHB (**b**), 200 mg/l PHB (**c**) and 250 mg/l PHB (**d**).

Production of the virulence factor haemolysin was inhibited around the wells of PHB-treated cell-free supernatant (CFS) of *Vibrio* PUGSK8. The haemolysin activity was reduced in the plates to which CFS collected from 24-h cultures of *Vibrio* PUGSK8 treated with PHB had been added (Figure 3B). Complete inhibition of haemolysin production was observed in plates to which CFS collected from 48-h cultures of *Vibrio* PUGSK8 treated with PHB had been added (Figure 3C). This inhibition of haemolytic activity (Figure 3C), provides direct evident that PHB controls the phenotypic expression of this virulence factor in the strain. PHB depolymerase activity in *Vibrio* PUGSK8 could be clear visualised on PHB agar plates (Figure 4). Degradation of PHB occurred in both minimal agar and Zobell marine agar (ZMA, Himedia, Mumbai, India) indicating that PHB depolymerase activity in *Vibrio* PUGSK8 does not appear to be dependent on the nutritional availability in the medium. The turbidometric assay showed that *Vibrio* PUGSK8 produced a maximum activity of 25.72 U/ml of PHB depolymerase at 48 h, with production of depolymerase increasing at the onset of stationary phase. ESI-HRMS analysis of enzyme hydrolysed PHB produces a peak at *m*/*z* 105.05, corresponding to the mass of butyric acids (Supplementary Figure S3).

### *N*-acyl-homoserine lactone degradation

**Figure 5:**
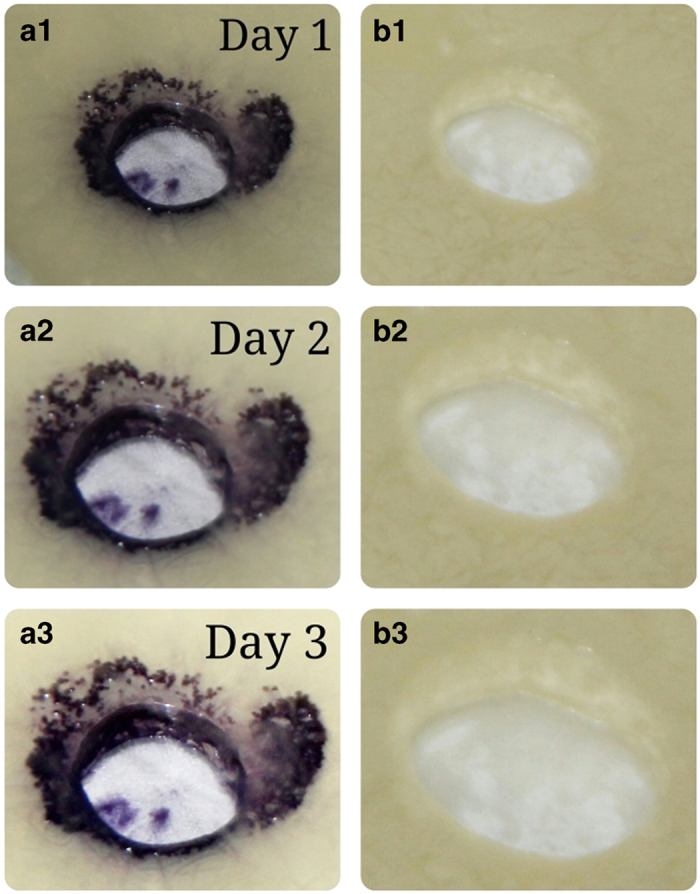
Effect of PHB on the degradation of AHL produced by *Vibrio* PUGSK8. The assay was performed on CV026 with AHL extract of *Vibrio* PUGSK8. a1, a2 and a3 show purple colour as an indicator of AHL expression around the wells filled with AHL extract of *Vibrio* PUGSK8. b1, b2 and b3 are showing loss of purple colour indicating AHL degradation by PHB (50 μg/ml) treated *Vibrio* PUGSK8 extract.

**Figure 6:**
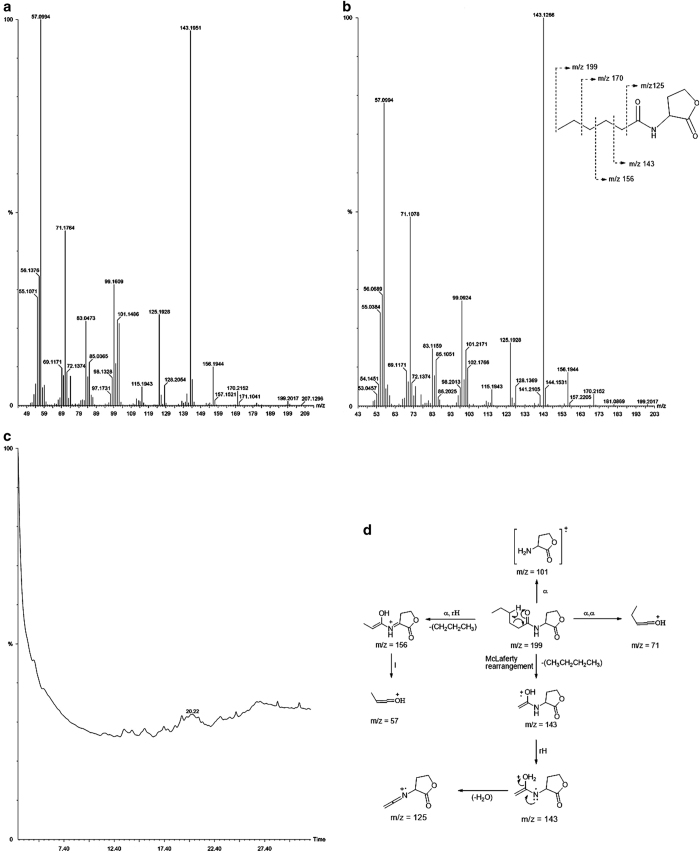
GC-MS analysis of AHL signalling molecules produced by *Vibrio* PUGSK8. (**a**) is showing GC-MS characteristic peaks of standard AHL. (**b**) Characteristic peaks of AHL produced by *Vibrio* PUGSK8 evidences the cell signalling molecule as *N*-hexanoyl homoserine lactone. (**c**) Absence of characteristic GC-MS peaks of AHL indicating the PHB shuts off PHB mediated cell signalling in *Vibrio.* (**d**). The fragmentation pattern elucidated based on the MS spectra of AHL produced by *Vibrio* PUGSK8 showed conformity with Cataldi *et al.* (2008).

*Vibrio* PUGSK8 appears to possess the ability to degrade AHL as evidenced by a loss in purple colour in the reporter strain *Chromobacterium violaceum* CV026 in the presence of extracts from PUGSK8 grown in the presence of 50 μg/ml PHB (Figure 5, b); with the effect being observed even after 72 h (Figure 5, b3). Purple colour was observed in CV026 in the presence of extracts from PUGSK8, which was not grown in the presence PHB (Figure 5, a1–3). To further study this apparent AHL degradation, following extraction of the AHL the fragmentation pattern analysis following TLC plates identified a unique fraction with an Rf value of 0.67, which when compared with an AHL standard (sigma) indicated that the fraction may contain a *N*-hexanoyl homoserine lactone signalling molecule. To confirm the chemical identity of the TLC fraction, mass spectral analysis was performed. The mass spectrum (MS) of the *Vibrio* PUGSK8 fraction closely matched the spectrum from a standard C6-AHL (Figure 6a), confirming the presence of a C6 HSL (*N*-hexanoyl homoserine lactone) compound (Figure 6b). Further analysis of some of these selected fragments indicate that the fragment ion at *m*/*z* 143 may be due to a McLafferty rearrangement, which is a typical carbonyl group having a hydrogen atom in the γ-position (Figure 6d). This rearrangement would give rise to an enolic fragment and an olefin loss of water from the ion giving rise to an *m*/*z* 143. The loss of the characteristic AHL peak in the GC-MS spectra in PHB-treated *Vibrio* PUGSK8 cultures is indicative of AHL degradation, which accompanied the loss in quorum-sensing signalling as evidenced by the loss in purple colour in the reporter strain *C. violaceum* CV026 (Figure 6c).

### PHB inhibition of *Vibrio* virulence in Artemia

**Figure 7:**
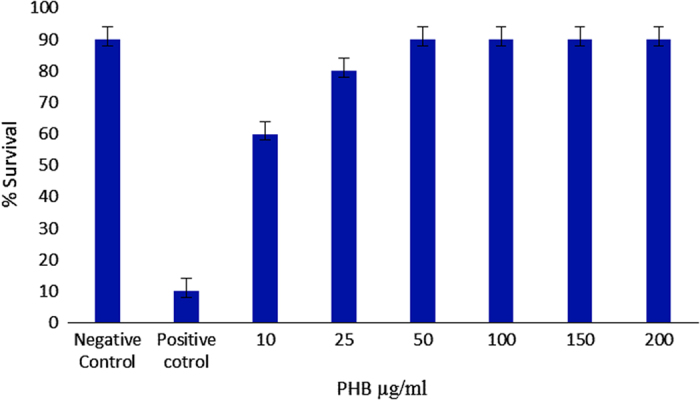
Effect of PHB and *Vibrio* on survival of Artemia. Positive control includes Artemia challenged with *Vibrio*. Negative control was Artemia without PHB treatment/*Vibrio* challenge.

PHB appears to exhibit anti-virulence effects *in vivo* and may regulate the phenotypic expression of virulence factors involved in the invasion of Artemia during *Vibrio* infections. PHB itself is not toxic to Artemia with the survival rate being unaffected at PHB concentrations between 50 and 200 μg/ml. In *in vivo* challenge experiments the treatment of Artemia with PHB at concentrations ⩾50 μg/ml appears sufficient to elicit complete protection against infections caused by pathogenic *Vibrio,* with protection being maintained up to 48 h post challenge. Lower survival rates were observed at lower PHB concentrations (10 and 25 μg/ml), with 60% and 80% survival, respectively, indicating that PHB at a concentration of 50 μg/ml was most effective in the Artemia model experiments (Figure 7). Conversely in the absence of PHB, survival rates in challenged Artemia started to decline after 6 h with mortality rates reaching 95% at 24 h, post challenge with *Vibrio*.

## Discussion

In this study, 27 luminescent *Vibrios* were isolated from the hepatopancreas of infected shrimp samples collected from shrimp farms located in southeast coast of India. From these 27 strains, the strain PUGSK8 was selected for target-specific assays based on its ability to produce high-intensity luminescence, which unlike some of the other isolates continued to be luminescent even after a number of sub-culturing steps. Luminescence is a part of the quorum-sensing system of *V. harveyi* and *V. campbelli* that is involved in the establishment of the pathogen in the host.^30,31^ Though bacteria from the *V. harveyi* clade and related bacteria are often referred to as luminescent *Vibrios,* a large difference between different strains with respect to luminescence has been reported.^32,33^ The non-luminescence we observed in the other strains may be due to defects in the autoregulation of the genes involved in the lux operon.^24^ Biochemical and morphological analysis of PUGSK8 indicated that it was from the *Vibrio* genus, while phylogenetic analysis showed a 99% match with *Vibrio campbellii* strains. *Vibrio* PUGSK8 exhibited flagellar and pili mediated motility, haemolytic activity, luminescence, together with the ability to form biofilms on glass, polystyrene and plastic. Pathogenic vibrios are the causative agent of vibriosis and are suspected to be involved in early-mortality syndrome or acute hepatopancreatic necrosis syndrome (EMS/AHPNS), which is a major thread to the shrimp aquaculture industry; causing 100% mortality, leading to the loss of >1 billion US dollars in recent outbreaks in many countries. Current treatment for *Vibrio* infections in shrimp involve reactive treatment with antibiotics and as with the use of many antibiotics poses the threat of the emergence of drug-resistant in vibrios. Indeed, antibiotics are becoming increasingly ineffective in the control of pathogenic vibrios and this coupled with the fact that the use of antibiotics in animal husbandry is banned in many countries; has resulted in an increased interest in the use of alternate treatment methods to treat *Vibrio* outbreaks in shrimp aquaculture. With this in mind we targeted the identification of anti-infective agents, which would interfere with the quorum-sensing system in luminescent *Vibrio* PUGSK8, which had been isolated from hepatopancreas of the infected shrimp *P. monodon*.

The non-specific surface adhesion behaviour of *Vibrio* PUGSK8 was evident and it exhibited a strong biofilm-forming potential. In this study, we used PHB produced by *Brevibacterium casei* MSI04 as a potential anti-infective molecule against luminescent *Vibrio* PUGSK8, as we had previously shown that PHB molecules are effective in the control of biofilm formation by vibrios.^17^ The microtitre-plate assay system coupled with the confocal laser scanning microscopy images revealed that PHB was very effective in the control/disruption of both biofilm formation and preformed biofilms. As in other *Vibrio* species the virulence cascade in *Vibrio* PUGSK8 includes motility behaviour, biofilm formation, quorum-sensing systems and haemolysin production. Thus, interfering with this virulence cascade in any way could prove an effective approach to help contain *Vibrio* infections in aquatic systems. The quorum-sensing system of *Vibrio* PUGSK8 is highly active and is likely to involve AHL molecules, given that the MS data showed characteristic peaks^34^ in extracts from the strain, which are similar to mass fragments of *N*-hexanoyl homoserine lactone in *Nitrosomonas europaea.*^35^ PHB effectively controls biofilm formation in *Vibrio* PUGSK8, the expression of bioluminescence, colonisation capacity and virulence cascade including motility and haemolysin, thereby reducing pathogenicity, and in doing so disrupts AHL-mediated quorum-sensing pathway. Given that the growth of *Vibrio* PUGSK8 remain unaffected by PHB, it appears likely that the observed effects may be as a result of PHB metabolism. A similar growth independent inhibition of quorum sensing has recently been reported for coumarin in *Pseudomonas aeruginosa.*^36^

It is well established that swarming motility and quorum sensing is necessary to develop colonial bacterial population both inside/outside the host.^37^ Motility behaviour and quorum-sensing molecules are important for cell differentiation, proliferation and sensing in bacterial populations. But in our findings it appears that in the PHB-treated *Vibrio* PUGSK8 the motility behaviour was completely reduced indicating the quorum-quenching nature of PHB. Recently, it has been reported that motility behaviour in *V. harveyi* is regulated through quorum sensing.^38^ Motility is also well established as a factor in the virulence cascade of pathogenic *V. harveyi*. As we have also demonstrated here in this study, inhibition of motility can significantly reduce virulence of *V. harveyi.*^38^

Bacteria within biofilms are highly resistant towards antibiotics.^39^ When the biofilm-forming capacity of bacteria is reduced, the resistance towards antibiotics and potential of pathogenesis is also reduced in the free-living *Vibrio* population. The experimental data generated here indicate that PHB may affect the pathogenicity of *Vibrio* by interfering with the signalling molecules. The quorum-quenching activity was revealed in the gas chromatography data, which showed no characteristic peaks/mass with respect to the AHL molecule. The reporter strain plate assay showed loss of characteristic colour formation in the PHB-treated plates of *Agrobacterium tumefaciens* and CV026. The quorum-quenching effect of PHB may be due to the hydrolysis of the PHB by PUGSK8 by secreting PHB depolymerase, which degrades the PHB into β-hydroxy butyric acid. This is the first report on luminescent *Vibrio* secreting PHB depolymerase, which was evident as a clear zone formation on the PHB plates. It has recently been reported that PHB at a concentration of 100 mg/l provides complete protection to the Artemia against *V. campbellii* infection.^29^ However, we report here that PHB at a concentration of 50 mg/l was effective in providing complete protection to Artemia against infection by *Vibrio* PUGSK8 in challenge experiments.

The bacteriostatic effect of short-chain fatty acids on *Enterobacteria*^40^ has previously been reported and may have promising effects on the control of bacterial diseases in aquaculture.^41^ PHB has previously been successfully used as an anti-infective in gnotobiotic studies with Artemia* franciscana.*^25,42^ The polymer PHB is not water soluble, and needs to be degraded into β-hydroxy butyrate monomers and oligomers in the gastrointestinal tract of the treated animals. Previous reports revealed that pretreatment of PHB with NaOH followed by digestion with gut enzymes increases the degradation of PHB.^43,44^ Degradation of PHB can also be achieved by PHA depolymerase producing bacteria and fungi.^45^ The mechanism of PHB degradation in treated animals, however, still remains unclear. It has been suggested that PHB may provide additional energy to act as an immunostimulant in the treated animals,^28^ or that PHB and its degradation products may lower the pH in the Artemia gut through non-ionic diffusion causing cellular acidification thereby inhibiting the virulence or pathogenesis of *Vibrio*. In contrast to this, the data presented here appear to indicate that the degradation intermediate of PHB regulates the phenotypic expression of the virulence cascade in luminescent *Vibrio* PUGSK8. Degradation of PHB by *Vibrio* PUGSK8 was observed on the media indicating that PHB depolymerase activity can inhibit the virulence cascade of *Vibrio*. The degradation intermediate of PHB was effective in inhibiting pathogen colonisation through biofilm formation and phenotypic expression of virulence factors. PHB exhibited a protective effect on Artemia against *Vibrio* infections.

Disruption of the quorum-sensing systems in *Vibrio* would be an effective and environmentally friendlier alternate to antimicrobial agents to control the pathogen. Thus, PHB treatments could be an eco-friendly non-invasive effectively strategy to contain *Vibrio* infection in shrimp aquaculture. It is well established that the treatment of individual infected aquatic animals is neither feasible nor possible, with entire system treatment approaches involving antibiotics culminating in resistant and residual impacts. The PHB-based treatment would be an effective anti-infective strategy to achieve aquatic system treatment approaches to contain *Vibrio* outbreaks in shrimp aquaculture. Although halogenated furanones have been shown to be effective in disrupting signalling molecules in Gram-negative bacteria,^46,47^ the administration of higher doses of furanones to shrimp larvae is both highly toxic and not effective in all the species and strains of *Vibrio* isolated from brine shrimp; thus, alternate molecules for treatments are required.^48^ An added advantage is that anti-infectives targeting quorum-sensing systems can be generated, which are target-specific and which are species or strain-specific, therefore minimising the possible development of resistance flora and the killing of untargeted microbial flora.

## Materials and methods

### Isolation and selection of pathogenic *Vibrio campbelli*

The pathogenic bacteria used in this study were isolated from the hepatopancreas of an infected shrimp collected from a shrimp hatchery in Kollam (8°54′ N 76°38′ E), which is located on the southwest coast of India. ZMA and nutrient agar with 2% NaCl was used for bacterial isolation, with pure cultures being obtained following incubation at 28 °C for 24 h. Selective isolation of *Vibrio* was performed on thiosulfate-citrate-bile salts-sucrose agar (Himedia). *Vibrio* cultures were examined for colony luminescence and intensity using a spectroflurometer (Fluorolog-FL3–11, Kyoto, Japan). DNA was isolated from PUGSK8 and PCR amplification of the 16S rRNA was performed using the primers 27F 5′-AGAGTTTGATCMTGGCTCAG-3′ and 1492R 5′-CGGTTACCTTGTTACGACTT-3′, generating a 1,500-bp size fragment. The amplified DNA was cloned using the TOPO TA cloning kit (Invitrogen, Carlsbad, CA, USA) for sequencing (Macrogen, Seoul, Korea). The forward and reverse sequences were obtained and compared for their pair wise similarity using the NCBI BLAST. Multiple alignments of these sequences were carried out by Clustal W 1.83 version of EBI (www.ebi.ac.uk/cgi-bin/clustalw/) with a transition weight of 0.5. Phylogenetic trees were constructed using MEGA 5.0 version (www.megasoftware.net) by means of the neighbour joining (NJ) and the unweighted pair group method along with the arithmetic mean (UPGMA) algorithms. The nucleotide sequences were deposited in Genbank with the accession number KR024645.

### Sources of PHB

The strain *B. casei* MSI04 (Genbank accession number KU510053) used for PHB production in this study was isolated from the marine sponge *Dendrilla nigra*, with its production, structural characterisation, anti-adhesive activity against *Vibrio* having previously being reported.^17^ Standard PHB (Sigma) was used for efficacy comparison in the preliminary experiments.

### Biofilm formation/disruption assay

The effect of PHB on biofilm formation in PUGSK8 was assessed in a 96-well microtitre-plate assay. Briefly, an overnight culture of *Vibrio* PUGSK8 was inoculated in 200 μl of Zobell marine broth in polystyrene microtitre plates. The test wells contained PHB at concentrations ranging from 50–150 μg/ml with wells without PHB and inoculum acting as positive and negative controls respectively. After 48-h incubation at 28 °C, the wells were washed twice with phosphate-buffered saline (pH 7.0) and adhered cells stained with 0.1% crystal violet and the amount of biofilm formation was quantified in a microplate reader (Labnics, Mumbai, India) at 590 nm.^17^ The biofilm-forming ability of *Vibrio* PUGSK8 was assessed with biofilms being allowed to develop on various surfaces such as glass, stainless steel, aluminium and polystyrene. Biofilms were stained with crystal violet and examined under a light microscope at ×40 magnification (Optika, Ponteranica, Italy). To visualise the effect of PHB on the biofilm-forming ability of *Vibrio* PUGSK8, biofilm was allowed to form on coverslips immersed in Zobell marine broth containing varying concentrations of PHB (50–150 μg/ml) and incubated at 28 °C for 24 h, with broth not containing PHB acting as a control. The coverslips were then washed with PBS, stained with 0.1% acridine orange and observed under confocal laser scanning microscopy (LSM 710, Carl Zeiss, Oberkochen, Germany).

### Motility assay

The motility behaviour of *Vibrio* PUGSK8 was evaluated using twitching, swimming and swarming plate assays.^49,50^ Swarm plates were prepared by adding 0.7% bacto agar to nutrient broth (Himedia), supplemented with 0.5% glucose and 100 μg PHB. Swimming assays were performed using tryptone swim agar plates containing 1% tryptone, 0.5% NaCl and 0.3% bacto agar supplemented with 100 μg PHB. Twitch agar plates were prepared by adding 1% tryptone broth, 5% yeast extract and 1% NaCl to LB broth supplemented with 1% bacto agar and 100 μg PHB. Plates without PHB addition served as controls and all plates were allowed to dry overnight at room temperature. Twitching plates were stab inoculated using a sterile tooth pick at a depth of 3 mm to the bottom of the petridish. Swarm plates were spot-inoculated on the agar surface. Swimming plates were seeded below the agar surface using a sterile inoculation needle. The plates were incubated at 37 °C for between 18 and 48 h. The diameters of swimming, swarming and twitching zones were measured and images were captured on a digital camera. (Nikon, Tokyo, Japan)

### Screening of quorum-sensing molecule secreted by *Vibrio* PUGSK8

#### Preparation of cell-free lysate for TLC

Reporter strains were cultured in LB broth containing kanamycin 20 μg/ml for *C. violaceum* CV026, and tetracycline 20 μg/ml for *A. tumefaciens*. *Vibrio* PUGSK8 was inoculated in ZMB, and all strains were incubated at 28 °C for 24 h. Broth cultures were centrifuged at 10,640*g* for 5 min. The supernatants were then passed through 0.1-μm Millipore filters (Merck) and the filtrate was extracted twice with an equal volume of acidified ethyl acetate. Pooled extracts were dried over anhydrous magnesium sulphate, evaporated to dryness and re-suspended in 50–100 μl of HPLC grade ethyl acetate. The presence of AHLs in the extracts was evaluated by C18 reverse-phase TLC plates (Merck)^51^ followed by fluorescence emission using a ultraviolet transilluminator. AHLs were identified by comparing the retention of synthetic standard AHLs (Sigma) and respective test AHL spots.

#### Detection of AHL by GC-MS

Extracts from *Vibrio* PUGSK8 and AHL standards (sigma) were analysed by gas chromatography–mass spectrometry (PerkinElmer-Clarus 680 model, Shelton, CT, USA) using the Elite 5MS column (PerkinElmer) (30 m×0.25 mm ID and 250-μm film thickness) operating in an electron impact mode at 70 eV with helium as the carrier gas at a flow rate of 1 ml/min. The mass spectral analysis was performed by comparing the mass values of AHL standards with the AHL of *V. campbellii.*

### Assessment of cell regulation and activity assays

#### Bacterial growth and luminescence

An overnight culture of *Vibrio* PUGSK8 was diluted to an OD 600 of 0.1 nm and inoculated into fresh ZMB containing 5 mg/100 ml PHB and incubated at 28 °C for 24 h. Flasks without the addition of PHB were used as controls. The growth and luminescence of *Vibrio* PUGSK8 was recorded after 6 h (OD 600 nm) at 1-h time intervals for 20 h using a spectrophotometer and spectroflurometer (Fluorolog-FL3–11). Growth and luminescence of *Vibrio* PUGSK8 in the presence of PHB produced by *B. casei* MSI04 was compared with that observed with the standard PHB (sigma).

### Haemolytic activity

Fifty-microlitre overnight *Vibrio* cultures grown in the presence of 0.5 μg PHB for 24 and 48 h, respectively, was centrifuged at 10,621*g* for 10 min and the CFS was filtred using 0.45-micron filtre. The assay was performed as described by Beecher and Wong^52^ with suitable modifications. Briefly, the CFS (75 μl) was added to well in a blood agar containing 5% (v/v) human blood and incubated at 37 °C for 24 h. After incubation, the plates were observed for the appearance of zones of haemolysis around the well. The CFS of cultures grown in the absence of PHB was used as a control.

### Detection of quorum-quenching activity using reporter strains and GC

The AHL inactivation assay was performed as described by Baruah *et al.*^53^ with necessary modifications. PUGSK8 was grown overnight in 100 ml ZMB supplemented with 50 μg/ml PHB and incubated at 28 °C for 48 h,^54^ with flasks without PHB addition serving as a control. Following centrifugation at 10,640*g* for 5 min the cell-free supernatant was separated and AHL was extracted, acidified with ethyl acetate and solvent evaporated to dryness. Reporter strain *C. violaceum* CV026 was grown overnight on LB broth and 100 μl of CV026 was swabbed onto the surface of LB agar plates. Wells were made using a sterile cork borer (0.6 mm diameter) on agar plates, and 10 mg/μl of extracted and air-dried *N*-hexanoyl homoserine lactone from PUGSK8 broth culture with PHB was added in triplicate to wells in the plates and incubated for 36–48 h. AHL extracted without PHB addition acted as controls. Plates were monitored for purple colour formation in the reporter strain CV026. Degradation of C6-HSL due to a lack of purple colour formation, was further verified using GC-MS; with extracts from *Vibrio* PUGSK8 grown in the presence of 50 μg/ml PHB being analysed.

### PHB degradation by *Vibrio* PUGSK8

To determine PHB degradation and utilisation by *Vibrio* PUGSK8, the degradation assay was performed as previously described with a modification to the growth media.^55^ *Vibrio* PUGSK8 was inoculated in nutrient rich (ZMA) medium as well as in the minimal media supplemented with various concentration of PHB ranging from 100 mg/l, 200 mg/l, 250 mg/l PHB. The plates were incubated at 28 °C for 48 h and monitored for the formation of zones of clearance. To determine the amount of PHB depolymerase produced by *Vibrio* PUGSK8, a turbidometric assay was performed as previously described^56,57^ Briefly, *Vibrio* PUGSK8 was cultured in minimal media supplemented with 0.1% PHB at 28 °C with agitation at 220 r.p.m. for different time intervals ranging from 12–48 h. The production media was centrifuged at 10,640*g* for 10 min. The crude enzyme extract was then vacuum filtered through 0.22 μm Millipore filtres. PHB depolymerase was then precipitated with equal volumes of cold acetone and the precipitate was dissolved in 10 mM acetate buffer pH 6.0. The precipitated enzyme was further purified by sephadex G-200 column chromatography. The active enzyme fractions were pooled and the enzyme assay was carried out. The assay mix was prepared with 0.7 mg of PHB in 1 ml of 10 mM acetate buffer with aliquots being mixed thoroughly using sonication for 10 min. The assay mix was added in triplicate to 0.5 ml of the enzyme solution and incubated at 40 °C for 20 min. The enzyme activity was calculated as a decrease in the turbidity at 660 nm with PHB without enzyme solution acting as a control blank. One unit of PHB depolymerase activity was defined as the amount of the enzyme solution (μl) to hydrolyse 1 μg of PHB per min. The degradation products formed resulting from depolymerase activity after 12 h of incubation was analysed Electrospray Ionisation-High Resolution Mass Spectral analysis (ESI-HRMS, Orbitrap Elite).

### Artemia assay

The *in vivo* challenge experiment was performed using a gnotobiotic model assay with *A. franciscana* as previously described.^53^ The experimental setup was performed in a sterile air laminar flow hood. Sterile cysts and larvae were obtained by decapsulation using 3.3 ml NaOH (32%) and 50 ml NaOCl (50%). Cysts were aerated in a 1-l capacity sterilised glass cylinder (jar) containing autoclaved seawater. To provide complete hydration of the cysts, an air stone was placed in the bottom of the jar seawater and oxygenated continuously using aerator pumps. After 24 h incubation at 28 °C the newly hatched free-swimming pink-coloured nauplii were collected from the bottom of the jar. The axenicity of the nauplii was confirmed by plating 50 μl of the hatched water onto ZMA plates and incubating at 28 °C for 7 days.

Preliminary dose selecting experiments were performed to find the effective concentration of PHB and to determine any toxic effect of PHB on Artemia. Newly hatched Artemia were exposed to various concentration of PHB ranging from 25 to 200 μg/ml. After exposure, the experimental setup was incubated at 28 °C under constant aeration and illumination. The survival of Artemia was recorded after 24 and 48 h. All experiments were performed in triplicate.

### Challenge experiment with gnotobiotic Artemia

Groups of 20 freshly hatched nauplii were transferred into sterile 50-ml beakers containing 25 ml sterilised seawater. The experiments were performed in triplicate with parallel negative control (Artemia with dried yeast 6 mg/ml), positive control (Artemia challenged with luminescent *Vibrio* PUGSK8 (10^5^ CFU/ml)) and test (Artemia treated with luminescent *Vibrio* PUGSK8 of 10^5^ CFU/ml along with 50 μg/ml of PHB). All experiments were performed in sterile conditions at 28 °C under light with continuous aeration. The survival rate was determined after 24 and 48 h by transferring the live Artemia to a watch glass and counting manually.
