# Supplementary material for: Degradation intermediates of polyhydroxy butyrate inhibits phenotypic expression of virulence factors and biofilm formation in luminescent Vibrio sp. PUGSK8
Source: NPJ Biofilms Microbiomes. 2016 Jun 15;2:16002–. doi: 10.1038/npjbiofilms.2016.2 (PMC5515267; doi:10.1038/npjbiofilms.2016.2)
Supplement: Supplementary Information [file npjbiofilms20162-s1.doc]

**
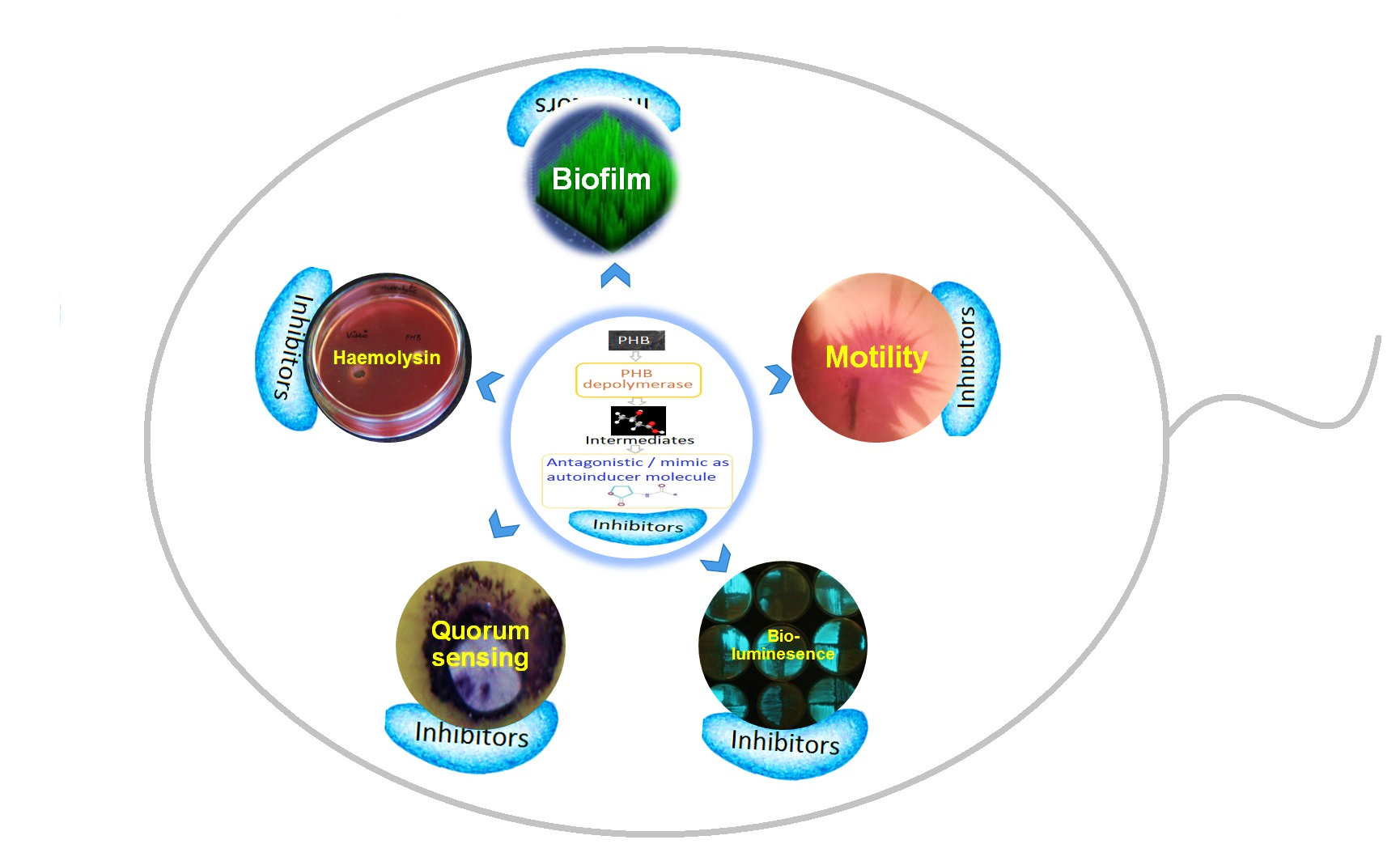
Striking image.** Postulated inhibition of virulence cascade by PHB degradation intermediates.

**Biofilm forming capacity of *Vibrio* PUGSK8**

**
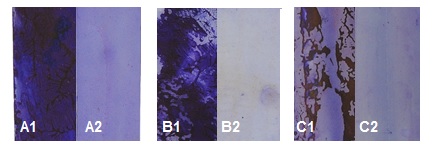
**

**Figure S1. Biofilm formed by the strain *Vibrio* PUGSK8**. Biofilm formed on various surfaces include glass, polystyrene and plastic. A1, B1 and C1 are *Vibrio* biofilm formed on glass, polystyrene and plastic surfaces and A2, B2 and C2 are controls without biofilm.

***In vivo* challenge experiments**

*Experimental Methods*: The *in vivo* challenge experiments were conducted in healthy shrimps reared in the aquarium facility. Asian tiger shrimp, *Penaeus monodon* seeds (post larvae, PL-20) was reared in 1 t capacity FRP tanks. The tanks were filled with aerated seawater and approximately 50% water exchange daily. Shrimp were fed with pelleted feed
(C.P. Grower feed No. 1) in three equal instalments per day. Optimum hydrological conditions was maintained with temperature of 30±2°C, pH of 7.8±0.5, dissolved oxygen content of 5.6±0.4 ppm. Healthy juveniles of 30 DOC were segregated and maintained at a rate of 10 shrimps per 100 l glass aquaria. Random samples of shrimp was dissected and bacterial isolation was performed on TCBS agar plants to ensure the shrimp were pathogen free. Overnight grown fresh shake culture of *Vibrio* PUGSK8 was centrifuged at 5000 g for 15 min and washed twice in normal saline (NS). The purified pellets were serially diluted in NS and enumerated in a Petroff-Hausser chamber. Then plated on TCBS agar to count colony forming units (cfu) per ml. The challenge dose was set between 10^2^ to 10^7^ cfu in 0.1 ml NS and inoculated intramuscularly using a 1 ml tuberculin syringe at ventral side between the second and third segment of healthy shrimps. The control group was set with 0.1 ml NS. Mortality and morbidity after challenge was monitored continuously for 24h and then every 12 h for 7 days. Moribund shrimps were sampled for reisolation of *Vibrio* PUGSK8 on TCBS agar plates.

*Experimental Results*

Based on the median lethal dose, the *Vibrio* PUGSK8 can be grouped under potential shrimp pathogen as the challenge showed the LD_50_ range at 10^3^ CFU/shrimp (Figure S1). The external symptoms was characterized as shell disease with necrosis on the shell, chelate legs, anorexia, feeble movement and mortality. The infected and moribund shrimps of every challenge were used for re-isolation. The luminescent *Vibrio* was re-isolated from infected shells, hepatopancrease and haemolymph.


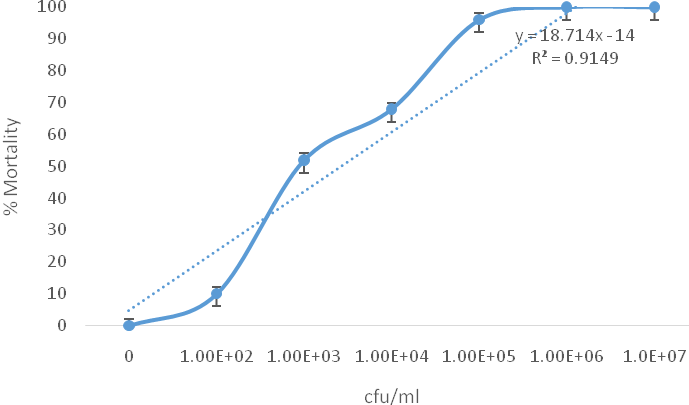


**Figure S2. Median lethal dose of *Vibrio* PUGSK8 challenge to *P. monodon***


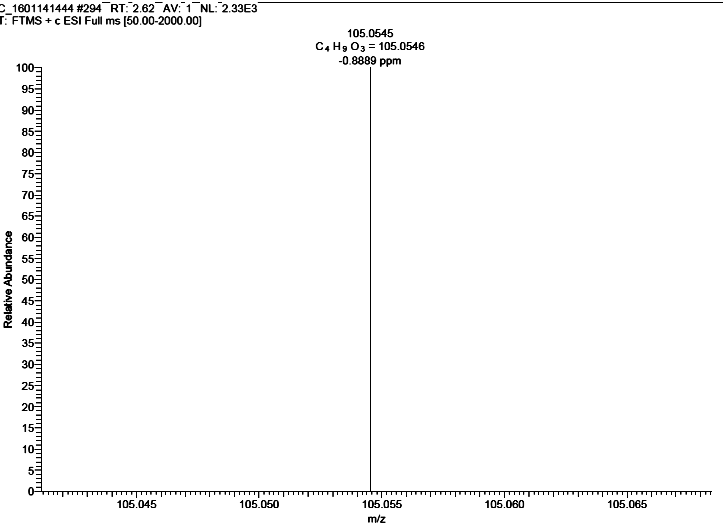


**Figure S3.** Enzyme hydrolysed products of PHB was analyzed through Electrospray Ionization-High Resolution Mass Spectral analysis (ESI-HRMS). Based on the ESI-HRMS results, the protonated species molecular ion peak was appeared at 105.0545 (M+1), which is matching the theoretical M+1 peak of butyric acid (m/z = 105.0546).

| Isolates | Gram staining | Resistance to ampicillin | Catalase | oxidase | Phospholipase | TPS | Hemolysin | Luminescence | Citrate | Urease | MR | VP | Motility |
| --- | --- | --- | --- | --- | --- | --- | --- | --- | --- | --- | --- | --- | --- |
| PU1 | G- curved rod | + | - | + | + | + | + | + | + | - | - | - | + |
| PU2 | G- curved rod | - | + | - | + | + | + | + | + | + | - | + | + |
| PU3 | G- curved rod | + | - | + | + | - | + | + | - | - | + | + | + |
| PU4 | G- curved rod | - | + | + | + | - | + | + | + | + | - | + | + |
| PU5 | G- curved rod | + | + | - | + | + | + | + | + | - | + | + | + |
| PU6 | G- curved rod | + | - | + | + | + | + | + | + | - | - | + | + |
| PU7 | G- curved rod | + | - | + | + | + | + | ++ | - | - | + | + | + |
| **PUGSK8** | G- curved rod | + | + | + | + | + | + | ++++ | - | - | + | - | + |
| PU9 | G- curved rod | - | + | + | + | - | + | + | - | - | + | + | + |
| PU10 | G- curved rod | + | - | + | + | - | + | ++ | + | - | + | + | + |
| PU11 | G- curved rod | + | + | - | + | + | + | + | + | - | + | + | + |
| PU12 | G- curved rod | + | + | + | + | + | + | + | + | - | + | + | + |
| PU13 | G- curved rod | + | + | + | + | + | + | +++ | + | - | + | + | + |
| PU14 | G- curved rod | - | - | + | + | - | + | + | + | - | + | + | + |
| PU15 | G- curved rod | + | - | + | + | - | + | ++ | + | - | + | + | + |
| PU16 | G- curved rod | - | - | + | + | - | + | + | + | - | + | + | + |
| PU17 | G- curved rod | + | + | + | + | + | + | + | + | - | + | + | + |
| PU18 | G- curved rod | - | - | + | + | - | + | + | + | - | + | + | + |
| PU19 | G- curved rod | + | + | + | + | + | + | + | - | - | + | + | + |
| PU20 | G- curved rod | + | + | + | + | - | + | + | - | + | + | - | + |
| PU21 | G- curved rod | + | + | - | + | + | + | + | + | - | + | + | + |
| PU22 | G- curved rod | + | + | + | + | + | + | + | - | + | + | - | + |
| PU23 | G- curved rod | + | - | + | + | - | + | ++ | + | - | - | + | + |
| PU24 | G- curved rod | - | + | + | + | + | + | + | + | - | + | + | + |
| PU25 | G- curved rod | + | + | - | + | + | + | + | - | - | + | - | + |
| PU26 | G- curved rod | + | + | + | + | + | + | + | - | + | + | - | + |
| PU27 | G- curved rod | + | - | + | + | - | + | ++ | + | - | + | + | + |

Table S1. Morphological and biochemical characteristics of the 27 luminescent colonies isolated moribund shrimp samples
